# Fractured Connections: Urban-Rural Divide in China Linking Elder Abuse, Social Networks, and Depression

**DOI:** 10.1093/geroni/igaf122.216

**Published:** 2025-12-31

**Authors:** Shibin Yan, Zi Yan, Xin Sun

**Affiliations:** University of Pennsylvania, Camden, New Jersey, United States; Waseda University, Tokyo, Tokyo, Japan; Fudan University, Shanghai, Shanghai, China (People’s Republic)

## Abstract

The contextual theory of elder abuse suggests that elder abuse emerges from interactions across individual, relational, community, and societal contexts. While prior research has established the negative effects of elder abuse on older adults’ physical and psychological well-being, its impact on their social dimensions—such as changes in social networks and the subsequent effects of those changes on their psychological well-being—remains underexplored. This study examines the relationship between elder abuse, social networks, and depressive symptoms using longitudinal data from 6,424 older adults (aged60 years and older) in the 2018 and 2020 China Longitudinal Ageing Social Survey. Mediation models were employed to assess whether changes in different types of social networks (contact, emotional, and instrumental) mediated the association between elder abuse experienced in 2018 and depressive symptoms in 2020. Mediation effects were estimated using Stata 17. Findings revealed that changes in social networks mediated the relationship between elder abuse and depressive symptoms, with notable urban-rural differences. Among rural older adults, elder abuse was linked to a decline in contact and emotional networks, which in turn exacerbated depressive symptoms. Conversely, among urban older adults, elder abuse was associated with an expanded instrumental network and lower depressive symptoms. These findings highlight the critical role of social networks in shaping the psychological outcomes of elder abuse victims. The divergent patterns between urban and rural older adults suggest the need for tailored social support interventions to mitigate the psychological impact of elder abuse.

